# Factors Associated with Chronic Kidney Disease in Patients with Type 2 Diabetes in Bangladesh

**DOI:** 10.3390/ijerph182312277

**Published:** 2021-11-23

**Authors:** Sheikh Mohammed Shariful Islam, Masudus Salehin, Sojib Bin Zaman, Tania Tansi, Rajat Das Gupta, Lingkan Barua, Palash Chandra Banik, Riaz Uddin

**Affiliations:** 1 Institute for Physical Activity and Nutrition, Deakin University, Burwood, VIC 3125, Australia; r.uddin@deakin.edu.au; 2 School of Health, Federation University Australia, Berwick, VIC 3806, Australia; masudussalehin@students.federation.edu.au; 3 Department of Medicine, School of Clinical Sciences at Monash Health, Monash University, Melbourne, VIC 3168, Australia; sojib.zaman@monash.edu; 4 Bangladesh College of Home Economics, University of Dhaka, Dhaka 1215, Bangladesh; tania.tansi.islam@gmail.com; 5 Arnold School of Public Health, University of South Carolina, Columbia, SC 29208, USA; rajatdas@email.sc.edu; 6 Department of Noncommunicable Diseases, Bangladesh University of Health Sciences, Dhaka 1216, Bangladesh; lingkanbarua@gmail.com (L.B.); palashcbanik@gmail.com (P.C.B.)

**Keywords:** type 2 diabetes mellitus, chronic kidney diseases, hypertension, risk factors, Bangladesh

## Abstract

Diabetes and chronic kidney disease (CKD) are a major public health burden in low- and middle-income countries. This study aimed to explore factors associated with CKD in patients with type 2 diabetes (T2D) in Bangladesh. A cross-sectional study was conducted among 315 adults with T2D presenting at the outpatient department of Bangladesh Institute of Health Sciences (BIHS) hospital between July 2013 to December 2013. CKD was diagnosed based on the estimated glomerular filtration rate using the ‘Modification of Diet in Renal Disease’ equations and the presence of albuminuria estimated by the albumin-to-creatinine ratio. Multivariate logistic regression analysis was used to determine the factors associated with CKD. The overall prevalence of CKD among patients with T2D was 21.3%. In the unadjusted model, factors associated with CKD included age 40–49 years (OR: 5.7, 95% CI: 1.3–25.4), age 50–59 years (7.0, 1.6–39), age ≥60 years (7.6, 1.7–34), being female (2.2, 1.2–3.8), being hypertensive (1.9, 1.1–3.5), and household income between 10,001 and 20,000 Bangladeshi taka, BDT (2.9, 1.0–8.2) compared with income ≤10,000 BDT. However, after adjustment of other covariates, only the duration of hypertension and household income (10,001–20,000 BDT) remained statistically significant. There is a need to implement policies and programs for early detection and management of hypertension and CKD in T2D patients in Bangladesh.

## 1. Introduction

Chronic kidney disease (CKD) has been recognized as a global public health burden with increasing prevalence and mortality [1]. In 2017, 1.2 million deaths were due to CKD worldwide [1]. In South Asia, the prevalence of CKD was reported as 13.6% in Thailand [2], 10.8% in China [3], and 15.0% in India [4]. CKD often leads to progressive end-stage renal disease (ESRD), increased hospitalization, and premature deaths [5]. The prevalence of CKD has been increasing in many low- and middle-income countries (LMICs) where there is a lack of health systems’ capacity for prevention and management. CKD is more prevalent among the aged population and is also a common complication for people with diabetes and hypertension. In South Asia, the increasing aging population and rapid urbanization have led to an increase in obesity and diabetes mellitus [6,7]. If this trend continues to rise, it will likely spike the proportion of people with diabetes at a faster rate over the next two decades [6]. Particularly, CKD related to type 2 diabetes (T2D) will rise in parallel [8]. 

Globally, the prevalence of diabetes has increased in many countries over the past 30 years and inevitably, there are proportionally more people with T2D and CKD now than 30 years ago [9]. Diabetes is a major cause of CKD, accounting for about 40% of new patients [10]. Large-scale epidemiologic surveys have identified several important risk factors for CKD, including old age, presence of T2D, female sex, low socioeconomic status, and use of herbal medicine [11]. Diabetes is a significant risk factor for accelerated progression of CKD towards ESRD, kidney failure, and renal replacement therapy [12] and is considered to be a major contributor to chronic vascular diseases. The presence of diabetes in people with CKD deteriorates cardiovascular events [13] and worsens the survival for patients with dialysis or who have undergone kidney transplantation [14].

In recent years, the prevalence of T2D and hypertension has grown rapidly and is expected to increase the prevalence of CKD at an epidemic rate in South Asia [8,15,16]. Moreover, CKD causes an increased risk of myocardial infarction, stroke, and cardiovascular-related mortality in patients with T2D in comparison with non-diabetic patients [17]. Early detection of diabetic patients with CKD is important for monitoring cardiovascular risk factors and initiating therapy, which can slow down the progression of kidney failure [18]. There have been several reports on the prevalence and risk factors of CKD in patients with T2D in developed countries [11,19]. However, studies on factors associated with CKD in T2D patients are scarce in some developing countries like Bangladesh. 

A recent systematic review found that the overall pooled prevalence of CKD among Bangladeshi adults was 17.3%, ranging from 12.8% to 26.0% [20]. Another study reported that people with type 2 diabetes in Bangladesh had a high proportion of self-reported kidney disease [21]. A number of studies in Bangladesh have also reported the prevalence of kidney disease in different population groups [22,23,24]. A few studies have reported on CKD in patients with T2D and its determinants in Bangladesh at community levels [25], in slums [26], in the rural population [27], or during screening [28]. However, information on CKD and its associated factors in T2D patients in urban hospitals is limited. Understanding the factors associated with CKD among people with T2D in urban hospital settings in Bangladesh is essential to develop preventive strategies and improve management practices. Therefore, the aim of this study was to determine the prevalence of CKD among patients with T2D and its associated factors in an urban hospital in Bangladesh. 

## 2. Materials and Methods

### 2.1. Study Design and Population

This cross-sectional analysis was based on the baseline data of the Mobile Phone Intervention for Diabetes (MPID) study in Bangladesh [29]. The data were collected from July 2013 to December 2013. Details on participant selection, assessment process, and clinical data measurement were described in the study protocol, which was published previously [30]. Briefly, the MPID was a single-center randomized controlled intervention trial to measure the effects of a six-month mobile phone text messaging intervention program on glycemic control in people with T2D. The inclusion criteria were adult patients with T2D attending the outpatient department of Bangladesh Institute of Health Sciences Hospital (BIHS), who owned a mobile phone, and were willing to receive a text messaging program on lifestyle behavior change for six months. The BIHS hospital serves more than 2 million people living in Dhaka city and the surrounding areas in Bangladesh. All potential eligible patients referred by an attending BIHS physician and providing written informed consent were screened for eligibility and baseline data and blood samples were collected. 

### 2.2. Data Collection and Process

All patients underwent a structured interview to collect information on socio-demographics, including age, sex, household income, and education; time of diabetes onset and duration; presence and duration of hypertension; status of fasting plasma glucose (FPG), two-hour post prandial glucose (2h PPG) and glycated hemoglobin (HbA1c); and blood pressure (BP) readings, and lipid-lowering therapy, with an indication of the type/class of drug/medication (antihypertensive, anti-dyslipidemia, oral antihyperglycemic agents, etc.). 

### 2.3. Measurements and Definitions

The body weight and height of the participants were measured to the nearest 0.1 kg and 0.1 cm, respectively, using a standard protocol [30] and were used to calculate the body mass index (BMI; in kg/m^2^). BP was measured three times by a trained physician using a digital sphygmomanometer (Omron, SEM-1, Omron Corp, Osaka, Japan) in a sitting position after patients had taken five minutes to rest and a five-minute interval between the records. The mean of the last two measures was used to define BP status. Hypertension was defined by systolic BP ≥140 mmHg and/or diastolic BP ≥90 mmHg and/or ongoing anti-hypertensive treatment. Glycated hemoglobin (HbA1c) was measured by high-performance liquid chromatography using Diabetes Control and Complications Trial (DCCT) aligned methods. Serum triglycerides (TGs), total cholesterol (TC), and high-density lipoprotein (HDL) cholesterol were determined by colorimetric enzymatic methods; LDL cholesterol was calculated by the Friedwald formula, i.e., LDL-cholesterol (in mmol/L) = TC − [HDL-cholesterol + (TG/2.17)]. Diabetes was defined according to WHO criteria as fasting plasma glucose values of ≥7.0 mmol/L (126 mg/dl), 2-h post-prandal plasma glucose ≥11.1 mmol/L (200 mg/dl), HbA1c ≥6.5% (48 mmol/L), or a random plasma glucose ≥11.1 mmol/L (200 mg/dl) in the presence of signs and symptoms of diabetes [31]. 

The presence of CKD was assessed using the most recent urinary albumin and serum creatinine reports extracted from the patient’s medical report during the last 12 months. We included reports where the tests were performed at the BIHS hospital to avoid measurement bias. The estimated glomerular filtration rate (eGFR) was calculated using the Modification of Diet in Renal Disease (MDRD) equations [32]. Patients with eGFR <60 mL/min/1.73 m^2^ present for three months or more and/or markers of kidney damage determined by the presence of albuminuria estimated by the albumin-to-creatinine ratio were defined as CKD according to The National Kidney Foundation’s Kidney Disease Outcomes Quality Initiative [33]. 

### 2.4. Data Analysis

Statistical analyses were conducted using SPSS 21 (IBM Corporation, Armonk, NY, USA). Continuous data were expressed as the mean ± standard deviation (SD), or median and interquartile ranges, and categorical data were presented as a number with percentages. The Kolmogorov–Smirnov test was used to check normality for the continuous variables. We compared the mean and median between groups using the t-test and Mann–Whitney U test, respectively. We used multiple logistic regressions to explore the factors associated with CKD. We identified factors that had a bivariate association with CKD at the 20% level of significance (*p* ≤ 0.20). We checked these variables for collinearity issues, and those without any collinearity were entered into the multivariable model. We expressed associations as an odds ratio (OR) with 95% confidence intervals (CIs). We considered a *p*-value of <0.05 as significant. 

### 2.5. Ethics

The study was conducted following the Declaration of Helsinki and the protocol was approved by the Research Review Committee and Ethical Review Committee of the International Center for Diarrhoeal Disease Research, Bangladesh (icddr,b). Written informed consent was obtained from all the participants. Permission was granted from the Director of BIHS hospital to use their facility for data collection.

## 3. Results

Overall, 500 patients were recruited, of which 185 (37%) had missing data on renal function and were excluded from the present analyses. Therefore, the analytical sample for this analysis was 315 participants with complete information on renal function as assessed by serum creatinine and albuminuria measurements. 

### 3.1. Characteristics of the Patients

The mean ± SD age of the patients was 50.7 ± 9.9 years. Patients with CKD were on average 4.6 years older than non-CKD patients (*p* < 0.001). CKD were more common in females with T2D than males: 54.3% and 68.7%, respectively. The median income of the patients with T2D was 35,000 BDT (equivalent to US$448.7). The education level of the majority of the patients with T2D (41.0%) was a higher secondary certificate or above. CKD was more common in patients with a secondary level of education (37.3%), and most non-CKD patients had a higher secondary level of education (44.4%) (Table 1). 

More than one-third (38.4) of the patients had diabetes for ≥5 years; however, CKD was more common (55.2%) among those who had diabetes for <5 years. The mean FPG was 7.6 ± 2.1 mmol/L among the T2D patients; however, the average FPG in CKD patients was about 0.6 mmol/L less than the non-CKD patients’ group. This study found that 2-h PPG was uncontrolled in the majority of patients with T2D (53.8%). Moreover, about half (45.9%) of the patients were in a poorly controlled glycemic state (HbA1c > 8%). Most of the T2D patients were hypertensive (60.0%). The duration of HTN was >5 years in the majority of T2D patients (54.6%) and CKD was more common among this group. Most of the patients with T2D were obese (87.8%). 

### 3.2. Prevalence of CKD

The overall prevalence of CKD among patients with T2D was 21.3%, which was higher among females (68.7%) compared to males (31.3%). FBS was optimal (<6.7 mmol/L) in most of the diabetic patients with CKD (47.7%). However, there was no significant difference in 2hABF in these patients. Around 97.0% of CKD patients with T2D were obese and 71.6% were hypertensive. CKD was more common in patients with a secondary level of education (37.3%) than patients who studied less than or higher than the secondary level.

### 3.3. Results of Bivariate Analysis

Table 2 presents the bivariable regression analysis of factors associated with CKD. Older patient groups had significantly greater chances of having CKD compared to the young patients group. Compared to patients aged <40 years, patients in the age group 40–49 years had more than a 5 times higher odds of developing CKD (*p* = 0.02) while for patients in the age group ≥60 years, the odds were 7.6 times higher (*p* = 0.01). Females had a 2.2 times higher odds of having CKD when compared with male patients (*p* < 0.01). This study found that the odds of developing CKD among hypertensive diabetic patients were higher than the normotensive diabetic patients (*p* = 0.03). However, the duration of diabetes (<5 or ≥5 years) was not significantly associated with CKD in our patient groups (*p* = 0.09).

### 3.4. Results of Multivariate Analysis

Table 3 presents the multivariate analysis of factors associated with CKD. We found only two factors that were statistically and significantly associated with CKD. Diabetic patients with a household income of 10,000–20,000 BDT had a higher odds of having CKD (OR = 4.12; 95% CI 1.12–15.20) than those with a lesser income. The duration of hypertension was found to be associated with a higher odds of CKD, i.e., with a one-year increase in the duration of hypertension among patients with T2D, the odds of developing CKD were 6% higher (OR 1.06; CI 1.00–1.12).

## 4. Discussion 

To the best of our knowledge, this is the first study that has estimated the prevalence of CKD and its associated factors in patients with T2D attending an urban hospital in Bangladesh. Almost one in five people with T2D in urban Bangladesh had CKD, which was almost twice as common in females than males. The key finding of our study is that household income and the duration of hypertension were significantly associated with CKD in our study population. The CKD prevalence in our study is similar to a previous study conducted in Thailand, where one-fifth of the participants with T2D attending a district hospital for diabetic care had CKD [34]. In Nepal, 20.3% of patients with T2D reported having CKD [35]. Studies conducted in other South Asian and developing countries reported a relatively higher prevalence. For example, CKD prevalence was 31.5% in Pakistan [36] and 24.7% in Tanzania [37]. However, our finding was in contrast with the DEMAND study, which assessed markers of CKD in 32,208 patients in a referred, clinic-based, or office-based setting across 33 countries and found ~50% of patients with T2D without a known history of proteinuria and/or diabetic kidney disease had CKD [38]. These discrepancies might be due to the differences in population health, geographic locations, methods of CKD diagnosis, quality of diabetes care, and available resources for management of T2D.

Hypertension is common in patients with CKD and those with T2D. Hypertension is a classical risk factor of kidney injury, and uncontrolled hypertension plays an important role in the progression of CKD by increasing glomerular filtration pressure and subsequent renal damage [39]. Patients with T2D may have hypertension for years prior to the onset of overt diabetes. Furthermore, people with diabetes have shown low medication adherence in Bangladesh, which exposes them to increased risks of diabetes complications [40,41,42]. Still, if BP rises further, these patients are subsequently likely to develop CKD and diabetic nephropathy. The community-based study of CKD among T2D patients in Taiwan showed that the presence and duration of hypertension were both significantly associated with proteinuria and renal impairment after controlling for covariates [43]. Apart from showing a higher prevalence of CKD in patients with hypertension (71.6%) in our study, the duration of hypertension among our population was also significantly associated with CKD after adjustment of all covariates, which is supported by the study of Thakur et al. in Nepal [35], which showed a significant association of high BP with microalbuminuria. Previous studies also showed a positive correlation between these two variables [44,45].

CKD is increasingly common in low- and middle-income countries, mostly due to a lack of healthcare infrastructure and resources for diagnosis and management, health insurance, and limited healthcare access [46]. Poverty is one of the most important markers of poor health and chronic diseases, including CKD [47]. Our results showed that participants with household incomes between BDT 10,001 and 20,000 had almost four times the odds of having CKD compared to those with household incomes <BDT 10,000. This might be due to the fact that low income groups avoid healthcare services as they cannot afford the high costs of treatment and also higher prevalence of hypertension among high-income groups in Bangladesh [48,49]. Our results contrast with a large registry-based study in India, where CKD was significantly lower in the highest income group [50].

Ageing is considered the most common cause of renal impairment in patients with T2D and with a global increase in life expectancy, the prevalence of CKD is expected to rise [51]. Our study found a non-significant association between aging and CKD. Previous studies have shown a higher prevalence of CKD in females [43,52], consistent with our findings. In our study, CKD was more prevalent (55.2%) in patients with diabetes <5 years, reflecting the early onset of CKD in patients with T2D, although the relationship was not statistically significant. However, a study in Nepal [35] reported a positive correlation between microalbuminuria and the duration (>5 years) of diabetes mellitus. Similar findings were observed by Idowu et al. and Varghese et al., who reported the duration of diabetes as a major predictor of microalbuminuria [53,54].

Our study had a number of limitations. First, we used a cross-sectional study design; therefore, we could not establish any causal relationship between CKD and its risk factors. Second, data were collected from a single hospital setting in Dhaka city and are not representative of the Bangladeshi population. Moreover, the study had a relatively small sample size, which may have limited the generalizability of the findings to other T2D patients in Bangladesh; hence, the results should be interpreted with caution. Third, data for this study were collected in 2013. There have been considerable changes in the sociodemographic and economic conditions of the country and the health behaviors of its population, which may have impacted on T2D and CKD in Bangladesh. Therefore, studies with more recent data are required. Fourth, urinary albumin and serum creatinine data were obtained from patients’ medical reports for tests conducted at the BIHS hospital only and thus, participants who tested in other facilities or did not have updated reports were excluded. Fifth, in the current study, other factors, such as history of atrial fibrillation, coronary artery disease, or heart failure, which may contribute to T2D and CKD, were not assessed. Finally, as with any cross-sectional study with self-reported data, recall bias or reporting bias could be a problem. Nevertheless, the findings of this study could shed light on future study design and more extensive studies with more robust measurements and follow-up are required.

Our study was conducted in an urban tertiary-level hospital, where one-fifth of the diabetic patients had CKD despite the availability of a wide range of healthcare services and facilities, for example, multidisciplinary specialists, counsellors, and research labs. This might also reflect the harsh scenario of the rural population in Bangladesh or similar country settings, where patients with T2D remain undiagnosed for an extended period of time and eventually present to the hospital when they have already developed macro- or microvascular complications [42,55]. Strategies for early detection of CKD and hypertension with innovative approaches to management are needed in patients with T2D in Bangladesh [56,57,58,59,60,61,62].

## 5. Conclusions

In conclusion, we found that more than one in five adults with T2D attending an urban hospital in Bangladesh had CKD. Higher household income and hypertension were associated with CKD in patients with T2D. There is an urgent need for collective efforts for the prevention, early detection, and management of hypertension and CKD in T2D patients in Bangladesh.

## Figures and Tables

**Table 1:** Basic characteristics of the study participants (*N* = 315).

Variables	*N* (%)
Age (years)	
Mean ± SD, years	50.7 ± 9.9
<40	44 (14.0)
40–49	103 (32.7)
50–59	104 (33.0)
≥60	64 (20.3)
Gender	
Male	144 (45.7)
Female	171 (54.3)
Household income (BDT)	
≤10,000 BDT	36 (11.8)
10,001–20,000 BDT	94 (30.8)
20,001–30,000 BDT	52 (17.0)
30,001–40,000 BDT	29 (9.5)
40,001–50,000 BDT	39 (12.8)
>50,000 BDT	55 (18.0)
Education	
No academic education	40 (12.7)
Primary (year 5 completed)	53 (16.8)
Secondary (year 10 completed)	93 (29.5)
Higher secondary and above (year 12 and above completed)	129 (41.0)
Duration of diabetes (years)	
Median (IQR) duration: 3.0 (1.0–7.0)	
<5 Years	194 (61.6)
≥5 Years	121 (38.4)
Duration of hypertension (years)	
Median (IQR) duration: 5.0 (2.0–10.0)	
<5 Years	83 (45.4)
≥5 Years	100 (54.6)
FPG (mmol/L)	
Mean ± SD.	7.6 ± 2.1
Optimal < 6.7 mmol/L	114 (36.9)
Fair 6.7–7.8 mmol/L	84 (27.2)
Poor > 7.8 mmol/L	111 (35.9)
2h AFB (mmol/L)	
Control < 10.0 mmol/L	141 (46.2)
Uncontrolled ≥ 10.0 mmol/L	164 (53.8)
HbA1c (%)	
Optimal < 7%	56 (28.9)
Fair 7–8%	49 (25.3)
Poor > 8%	89 (45.9)
Obesity	
Not obese	7 (2.2)
Obese	308 (97.8)
Hypertension	
Absent	126 (40.0)
Present	189 (60.0)

*Note* FPG: Fasting Plasma Glucose; BDT: Bangladeshi Taka, 10,000 BDT = $128.2 [1$ = 78 BDT]; IQR: interquartile range; HbA1c: Glycated Hemoglobin; 2 h ABF: Blood sugar 2 h after breakfast.

**Table 2:** Bivariate results of factors associated with CKD in T2D patients (*N* = 315).

Variables	CKD (*n* = 67) *n* (%)	Non-CKD (*n* = 248) *n* (%)	Unadjusted OR (95% CI)	*p*-Value
Age (years)				
<40	2 (3.0)	42 (16.9)	Reference	
40–49	22 (32.8)	81 (32.7)	5.7 (1.3–25.4)	0.02
50–59	26 (38.8)	78 (31.5)	7.0 (1.6–30.9)	0.01
≥60	17 (25.4)	47 (19.0)	7.6 (1.7–34.8)	0.01
Gender				
Male	21 (31.3)	123 (49.6)	Reference	
Female	46 (68.7)	125 (50.4)	2.2 (1.2–3.8)	0.01
Household income (BDT)				
≤10,000 BDT	5 (7.8)	31 (12.9)	Reference	
10,001–20,000 BDT	30 (46.9)	64 (26.6)	2.9 (1.0–8.2)	0.04
20,001–30,000 BDT	9 (14.1)	43 (17.8)	1.3 (0.4–4.3)	0.67
30,001–40,000 BDT	5 (7.8)	24 (10.0)	1.3 (0.3–5.0)	0.71
40,001–50,000 BDT	9 (14.1)	30 (12.4)	1.9 (0.6–6.2)	0.31
>50,000 BDT	6 (9.4)	49 (20.3)	0.8 (0.2–2.7)	0.67
Education				
No academic education	7 (10.4)	33 (13.3)	Reference	
Primary	16 (23.9)	37 (14.9)	2.0 (0.7–5.6)	0.17
Secondary	25 (37.3)	68 (27.4)	1.7 (0.7–4.4)	0.25
Higher secondary and above	19 (28.4)	110 (44.4)	0.8 (0.3–2.1)	0.67
Duration of diabetes (years)				
<5 Years	37 (55.2)	157 (63.3)	Reference	
≥5 Years	30 (44.8)	91 (36.7)	1.4 (0.8–2.4)	0.23
Duration of hypertension (years)				
<5 Years	18 (35.3)	65 (49.2)	Reference	
≥5 Years	33 (64.7)	67 (50.8)	1.8 (0.9–3.5)	0.09
FPG (mmol/L)				
Optimal < 6.7 mmol/L	31 (47.7)	83 (34.0)	Reference	
Fair 6.7–7.8 mmol/L	18 (27.7)	66 (27.0)	0.7 (0.4–1.4)	0.35
Poor > 7.8 mmol/L	16 (24.6)	95 (38.9)	0.5 (0.2–0.9)	0.02
2-h PPG (mmol/L)				
Control < 10.0 mmol/L	32 (50.8)	109 (45.0)	Reference	
Uncontrolled ≥ 10.0 mmol/L	31 (49.2)	133 (55.0)	0.8 (0.5–1.4)	0.42
HbA1c (%)				
Optimal < 7%	12 (36.4)	44 (27.3)	Reference	
Fair 7–8%	9 (27.3)	40 (24.8)	0.8 (0.3–2.2)	0.70
Poor > 8%	12 (36.4)	77 (47.8)	0.6 (0.2–1.4)	0.21
Obesity				
Not obese	2 (3.0)	5 (2.0)	Reference	
Obese	65 (97.0)	243 (98.0)	0.7 (0.1–3.5)	0.64
Hypertension				
Absent	19 (28.4)	107 (43.1)	Reference	
Present	48 (71.6)	141 (56.9)	1.9 (1.1–3.5)	0.03

*Note* CI: confidence interval; OR: odds ratio; FPG: Fasting Plasma Glucose; 2-h PPG: 2 h post prandal glucose; HbA1c: glycated hemoglobin; BDT: Bangladeshi Taka, 10,000 BDT = $128.2 [1$ = 78 BDT].

**Table 3:** Multivariate analysis of factors associated with CKD in T2D patients.

Variables	OR (95% CI)	*p*-Value
Age (years)		
<40	Reference	
40–49	2.82 (0.52–15.32)	0.231
50–59	2.50 (0.45–13.96)	0.295
≥60	2.51 (0.40–15.70)	0.327
Gender		
Male	Reference	
Female	1.76 (0.78–3.97)	0.177
Household income (BDT)		
≤10,000 BDT	Reference	
10,001–20,000 BDT	4.12 (1.12–15.20)	0.033
20,001–30,000 BDT	2.06 (0.48–8.88)	0.331
30,001–40,000 BDT	1.19 (0.21–6.65)	0.842
40,001–50,000 BDT	1.43 (0.31–6.62)	0.649
>50,000 BDT	0.71 (0.15–3.38)	0.664
Duration of hypertension (years)	1.06 (1.00–1.12)	0.049
FPG (mmol/L)	0.97 (0.81–1.17)	0.763
Hypertension		
Absent	Reference	
Present	1.56 (0.56–4.35)	0.392

*Note* CI: confidence interval; OR: odds ratio; FPG: Fasting Plasma Glucose; BDT: Bangladeshi Taka, 10,000 BDT = US$128.2 [1 US$ = 78 BDT]. Bold face indicates significance at 5% (*p* < 0.05) level.

## Data Availability

The data presented in this study are available on request from the corresponding author.
